# Tunable Fungal Monofilaments from Food Waste for Textile Applications

**DOI:** 10.1002/gch2.202300098

**Published:** 2023-09-17

**Authors:** E. R. Kanishka B Wijayarathna, Ghasem Mohammadkhani, Farshad Homayouni Moghadam, Linn Berglund, Jorge A. Ferreira, Karin H. Adolfsson, Minna Hakkarainen, Akram Zamani

**Affiliations:** ^1^ Swedish Centre for Resource Recovery University of Borås Borås SE‐501 90 Sweden; ^2^ Department of Animal Biotechnology, Cell Science Research Center, Royan Institute for Biotechnology ACECR Isfahan 83431 Iran; ^3^ Department of Engineering Sciences and Mathematics Luleå University of Technology Luleå SE‐971 87 Sweden; ^4^ Department of Fiber and Polymer Technology KTH Royal Institute of Technology Stockholm SE‐100 44 Sweden

**Keywords:** food waste, fungal textiles, hydrogel, tunable material, wet‐spinning

## Abstract

A fungal biorefinery is presented to valorize food waste to fungal monofilaments with tunable properties for different textile applications. *Rhizopus delemar* is successfully grown on bread waste and the fibrous cell wall is isolated. A spinnable hydrogel is produced from cell wall by protonation of amino groups of chitosan followed by homogenization and concentration. Fungal hydrogel is wet spun to form fungal monofilaments which underwent post‐treatments to tune the properties. The highest tensile strength of untreated monofilaments is 65 MPa (and 4% elongation at break). The overall highest tensile strength of 140.9 MPa, is achieved by water post‐treatment. Moreover, post‐treatment with 3% glycerol resulted in the highest elongation % at break, i.e., 14%. The uniformity of the monofilaments also increased after the post‐treatments. The obtained monofilaments are compared with commercial fibers using Ashby's plots and potential applications are discussed. The wet spun monofilaments are located in the category of natural fibers in Ashby's plots. After water and glycerol treatments, the properties shifted toward metals and elastomers, respectively. The compatibility of the monofilaments with human skin cells is supported by a biocompatibility assay. These findings demonstrate fungal monofilaments with tunable properties fitting a wide range of sustainable textiles applications.

## Graphical abstract

Tunable fungal monofilaments are produced by valorizing food waste using a biorefinery concept. By performing different post‐treatments stronger fibers with tensile strength of 140 MPa and flexible fibers with 14% elongation are produced. Finally, the produced monofilaments are compared with commercial fibers using Ashby's plots with a discussion of potential applications.

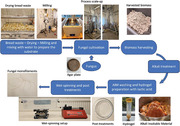

## 1 Introduction

The textile and fashion industry has grown to reach a status of uncontrollable environmental and ethical emergency.^[^ ^1^ ^]^ Due to the complicated supply chain and high energy consumption, larger than aviation and shipping combined, the fashion industry's contribution to the global greenhouse gas emissions is ≈10%.^[^ ^2^ ^]^ Fast fashion that enforced frequent consumption of cheaply manufactured garments with a short life span produces more than 92 million tons of waste annually.^[^ ^3^ ^]^ Biopolymers, the polymers synthesized by biological means such as nanocellulose, animal proteins, chitin, and chitosan are heavily researched and will play a key role in making the fashion industry more sustainable in the future.^[^ ^4^ ^]^

Chitin, the second most abundant biopolymer after cellulose, and chitosan, the deacetylated derivative of chitin are two biopolymers with potential for wide variety of applications such as tissue engineering, drug delivery, packaging, and textiles.^[^ ^5^, ^6^ ^]^ Mainly commercial chitosan is extracted from the crustaceans, and the extraction process creates harmful effluents containing acids and bases, which are utilized for demineralization, deproteinization, and especially deacetylation. Moreover, commercial chitosan production is not enough to meet the industry requirements, thus, alternative sources such as fungal chitosan are in high demand.^[^ ^7^ ^]^ When compared to crustacean chitosan, fungal chitosan is more environmentally beneficial and cost effective as the usage of strong and harmful chemicals is not required.^[^ ^8^, ^9^ ^]^ Moreover, zygomycetes fungi can deacetylate chitin to chitosan naturally by themselves^[^ ^10^ ^]^ which makes it more favorable when compared to economic and ecological aspects. Therefore, fungal chitin and chitosan have attracted a considerable amount of research interest throughout the past decade.

Filamentous fungi are widely used for bioconversion of food waste into value added products such as ethanol, lactic acid, food or feed, and pigments.^[^ ^11^, ^12^, ^13^, ^14^, ^15^ ^]^ Food waste has become the third‐highest greenhouse gas emitter globally with ≈931 million tons of food being wasted in 2019.^[^ ^16^ ^]^ Bread waste contributes to a significant fraction of food waste. Global bread production is more than 100 million tons in a year and ≈10% of that is wasted.^[^ ^17^ ^]^ Due to the large quantity of waste fraction and availability of the nutrients^[^ ^18^ ^]^ bread waste is extensively researched in biotechnological applications.

Filamentous fungithat belong to zygomycetes (as per the previous taxonomic classification), such as *Rhizopus delemar*, can naturally synthesize chitosan in the cell wall. The fungus is classified as mucoromycete in the latest fungal classification.^[^ ^19^ ^]^ According to their analysis of *Rhizopus delemar* cell wall using lytic enzymes, Tominaga and Tsujisaka^[^ ^20^ ^]^ confirmed that the cell wall contains chitin fibers cemented with chitosan and proteins. Moreover, the amount of chitin and chitosan in the cell wall varies with the growth conditions. In recent works, Svensson, Ferreira^[^ ^21^ ^]^ and Svensson, Oliveira^[^ ^22^ ^]^ have revealed that the chitin and chitosan amounts in *Rhizopus delemar* are ≈20% and 35% of the fungal cell wall, respectively. Tan, Tan^[^ ^10^ ^]^ have also shown that zygomycete fungus *Rhizopus oryzae* has a chitosan yield of ≈20% and several other fungi in the same phylum contain 15% to 25% chitosan in the cell wall. The whole biomass or cell wall fraction of the fungus *Rhizopus delemar* grown on bread waste has been used to make biobased materials such as fibers, films, and leather substitutes in recent research work.^[^ ^21^, ^22^, ^23^, ^24^ ^]^ Using plasticizers such as glycerol to reduce the brittleness in biobased materials was investigated in our previous research^[^ ^24^ ^]^ as well as by Appels, van den Brandhof.^[^ ^25^ ^]^

The goal of this research was to explore the possibility of tuning the properties of wet spun fungal monofilaments by introducing different post‐treatments. A wet spinning technique was used initially with ethanol as the coagulation bath to fabricate the fungal cell wall polymers into monofilaments. The hypothesis of tensile property enhancement from the water‐induced hydrogen bonding was tested. This phenomenon takes place when rehydrating a dried material due to the co‐polymeric action of water molecules.^[^ ^26^ ^]^ In another post‐treatment glycerol was used to incorporate the plasticizing effect to the produced monofilaments.^[^ ^25^ ^]^ The morphology and mechanical properties of the produced monofilaments after post‐treatments were examined. A biocompatibility test was done to investigate the compatibility of the monofilaments with human skin fibroblasts, as one potential application is to be used in textile. The mechanical data was analyzed using Ansys Granta EduPack software with the help of Ashby plots^[^ ^27^ ^]^ to find out where the monofilaments are placed in the material universe. This work unveils monofilaments with different properties along with diversified range of potential end uses.

## 2 Results and Discussion

Biobased materials tunable for different applications, such as the biobased monofilaments developed here from fungal cell wall, are on high demand considering the current urge on global sustainability.^[^ ^28^ ^]^ The fungal cultivation was done using bread waste as substrate and the harvested biomass then underwent an alkali treatment to extract the fungal cell wall. The extracted material was mechanically homogenized by ultrafine grinding and wet spun into monofilament yarns. The obtained monofilaments were subjected to different post‐treatments to tune the properties and the final characterization shows that the produced monofilaments are suitable for a wide range of applications.

### 2.1 Fungal Cultivation of Bread Waste and Preparation of Alkali Insoluble Material

*Rhizopus delemar* is known as a robust fungus and has been used in previous research to valorize bread waste with proven presence of the chitin and chitosan in the cell wall.^[^ ^22^, ^29^ ^]^ The fungal cultivation for this research was carried out for 48 h and the harvested biomass concentration was 6 ± 0.4 g L^−1^ from a 4% bread suspension. The biomass was subjected to an alkali treatment to separate the fibrous cell wall fraction. After the treatment, the alkali insoluble material (AIM), which is the fibrous cell wall fraction, was filtered and washed until neutral pH. The washed AIM, after pH adjustment to 3.5 using lactic acid, was behaving like a hydrogel due to the protonation of amino groups in cell wall chitosan.

### 2.2 Mechanical Treatment of Alkali Insoluble Material

**Figure 1:**
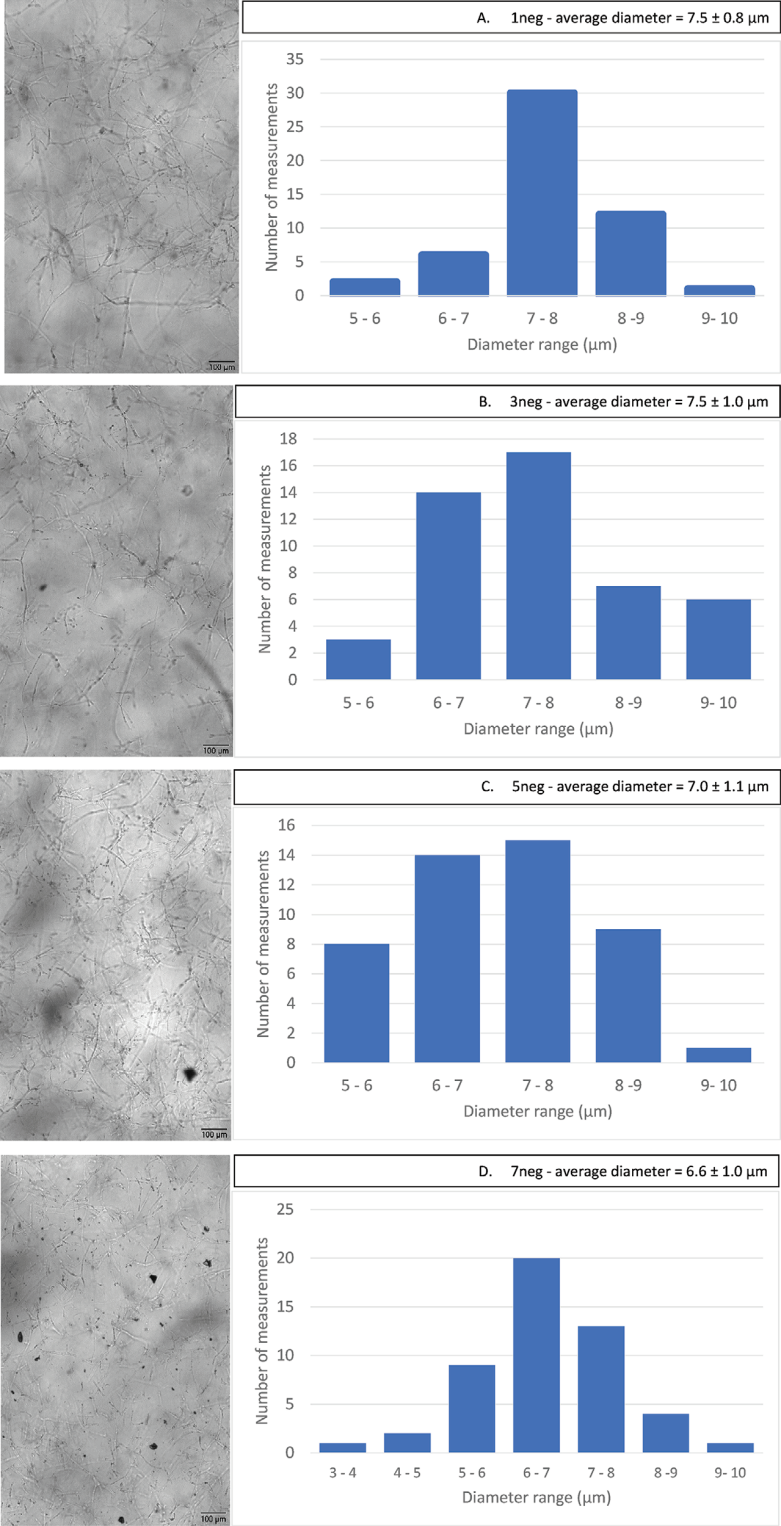
Images of fungal cell wall microfibers after different number of grinding cycles obtained from oCelloScope and the microfiber diameter distribution.

The concentration of gel obtained after pH adjustment was low and direct spinning was not possible, additionally the gel was not homogenous and contained visible clumps. To increase the homogeneity, the material was passed through the ultra‐fine grinder. Samples were collected for spinning dope preparation after different number of cycles through the grinder as mentioned in **Table** **1**. A microscopic analysis was done on the ground alkali insoluble material (AIM) using oCelloScope to estimate the size of the cell wall microfibers. According to **Figure** **1**A–C there is no noticeable change in the microfiber diameter thus it can be considered that homogenization has taken place, rather than a physical size reduction of the material. However, there is a clear difference in the fiber diameter after 7 grinder cycles (7neg). This further explains the tensile strength increment and elongation % at break decrement for the 7neg fibers (see **Table** **2**), which made the fungal monofilaments stronger but brittle due to the closely packed thin microfibers. The number of grinder cycles was further increased to 9, however, the spinning of monofilaments was not possible as the spun hydrogel disintegrated in the ethanol coagulation bath. Therefore, further investigations were not performed on those materials.

**Table 1:** Abbreviation and information of different sample types.

Sample	Grinding cycles	Coagulation bath	Post‐treatment	Diameter (mm)
1neg E	1. negative gap (‐100 µm)	Ethanol	–	0.20 ± 0.02
3neg E	3. negative gap (‐100 µm)	Ethanol	–	0.20 ± 0.04
5neg E	5. negative gap (‐100 µm)	Ethanol	–	0.20 ± 0.03
7neg E	7. negative gap (‐100 µm)	Ethanol	–	0.20 ± 0.01
1neg E W	1. negative gap (‐100 µm)	Ethanol	Water	0.18 ± 0.01
3neg E W	3. negative gap (‐100 µm)	Ethanol	Water	0.18 ± 0.03
5neg E W	5. negative gap (‐100 µm)	Ethanol	Water	0.19 ± 0.05
7neg E W	7. negative gap (‐100 µm)	Ethanol	Water	0.19 ± 0.05
7neg E 1% G	7. negative gap (‐100 µm)	Ethanol	1% Glycerol	0.18 ± 0.06
7neg E 2% G	7. negative gap (‐100 µm)	Ethanol	2% Glycerol	0.21 ± 0.04
7neg E 3% G	7. negative gap (‐100 µm)	Ethanol	3% Glycerol	0.18 ± 0.03

**Table 2:** Mechanical properties of the produced fungal monofilament yarns.

Sample	Tensile strength (MPa)	Elongation % at break	Young's modulus (MPa)
1neg E	46.5 ± 0.1	4.1 ± 1.1	1815 ± 347
3neg E	60.1 ± 6.1	3.9 ± 1.2	2941 ± 141
5neg E	60.9 ± 5.0	7.1 ± 1.4	2578 ± 332
7neg E	65.0 ± 5.4	4.0 ± 0.1	3286 ± 110
1neg E W	117.8 ± 2.5	4.9 ± 0.4	6383 ± 200
3neg E W	122.4 ± 2.8	2.6 ± 0.5	6947 ± 439
5neg E W	129.3 ± 0.8	2.9 ± 0.5	6993 ± 630
7neg E W	140.5 ± 2.9	2.9 ± 0.1	7590 ± 125
7neg E 1% G	87.0 ± 8.7	7.2 ± 0.8	3311 ± 150
7neg E 2% G	47.6 ± 3.2	11.6 ± 3.3	1588 ± 121
7neg E 3% G	30.8 ± 2.6	13.8 ± 2.2	427 ± 270

**Figure 2:**
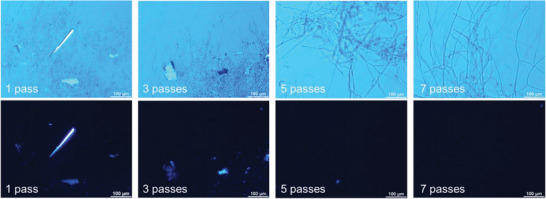
Polarized optical microscopy images of the fungal hydrogel samples after different number of cycles through ultrafine grinder. (1 cycle – 1neg, 3 cycles – 3neg, 5 cycles – 5neg and 7 cycles – 7neg).

The polarized optical microscopy images (**Figure** **2**) show how the homogeneity of the AIM increased with the different grinder cycles. Some larger unknown particles that could be substrate residuals and agglomeration of cell wall microfibers could be identified in 1 and 3 cycles samples. However, after 5 cycles the agglomeration became very low and after 7 cycles the sample does not contain any microfiber aggregation thus, the microscopic image shows a uniform dark image. This confirms that the sample contains well dispersed cell wall microfibers. In the blue images, the sample after 7 cycles shows the dispersed cell wall microfibers that are still in microscale. This confirms that only homogenization and opening up the microfiber agglomerates takes place in the ultrafine grinder. In their studies Svensson, Oliveira^[^ ^22^ ^]^ and Zhou, Sethi^[^ ^30^ ^]^ have shown similar dispersion behavior for fungal cell wall material and carrot nanofibers in different solvents, respectively.

### 2.3 Spinning of Fungal Monofilaments

The alkali insoluble material (AIM) that contains chitosan exhibits gelling behavior and homogeneity due to the protonation of amino groups in the glucosamine units in acidic pH^[^ ^22^, ^31^ ^]^ which facilitates the diffusion of water molecules through the chitosan chains. However, when water was used to dilute the gel to avoid clogging the membrane during the gel concentration, a drastic decrease in homogeneity and material precipitation was observed. This could be due to the deprotonation of the amino groups when the pH is increased from acidic to near neutral. Botelho da Silva, Krolicka^[^ ^32^ ^]^ have shown similar results in commercial chitosan hydro gelling as a result of the pH change. Consequently, the dilution was carried out using 0.5 M lactic acid to maintain the acidic pH that helped to retain the gelling behavior. The concentration of the hydrogel material after the vacuum funnel was between 5%–6 wt%. These hydrogels were successfully spun in the wet spinning setup.

**Figure 3:**
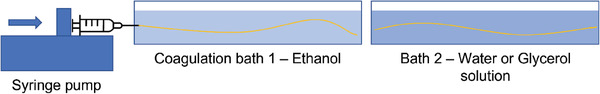
Schematic representation of the wet‐spinning and post‐treatment setup.

The hydrogels were wet spun using a simple lab scale extruder pump (New Era Pumps, USA) with a syringe and coagulation bath (**Figure** **3**). The material was directly extruded into ethanol where coagulation took place. Both chitin and chitosan which are available in the hydrogel are insoluble in ethanol; on the other hand, water and ethanol are completely miscible and form strong hydrogen bonding. Therefore, precipitation of as spun material takes place in ethanol due to phase inversion, thus a stable continuous monofilament is formed.^[^ ^33^, ^34^ ^]^ The fibers were kept in the coagulation bath for 2 min of retention time. During this time water molecules make stronger hydrogen bonds with ethanol^[^ ^35^ ^]^ resulting in dewatering of material and formation of a stable monofilament. Then the monofilaments were collected with laboratory tweezers and placed between magnets to dry. After overnight drying the monofilaments were collected. Post‐treatments were designed to test the tuneability of mechanical treatments. First, the plan was to investigate if use of glycerol in different percentages exhibits plasticizing effect^[^ ^25^ ^]^ and enhance flexibility of monofilaments. However, as a control, only water bath was also used. All the monofilaments when collected from the post treatment baths, showed an excessive increment of wet stretchability thus, before fixing them to the magnetic clamps for drying, ≈5% drawing was performed with the help of the clamping magnets (Figure S1). The monofilaments from glycerol treatments were softer when taken out from the bath therefore, the handling was more difficult compared with the monofilaments from only water post treatment. As the last step, all the monofilaments were left on the board with holding magnets at room temperature, until they were completely dried. The monofilaments post treated only with water had more even and uniform surfaces than the monofilaments which were not subjected to any post treatment. Monofilaments treated with glycerol were more stretchable with a moderately uniform surface. In our previous work vegetable tannin treated fungal sheets were dipped in a bath containing glycerol to reach elastic properties.^[^ ^24^ ^]^ Furthermore, Appels, van den Brandhof^[^ ^25^ ^]^ and Tarique, Sapuan^[^ ^36^ ^]^ have also used the plasticizing effect of glycerol in reaching elastic properties in films prepared from mushrooms and starch, respectively.

### 2.4 The Morphology of Monofilaments

**Figure 4:**
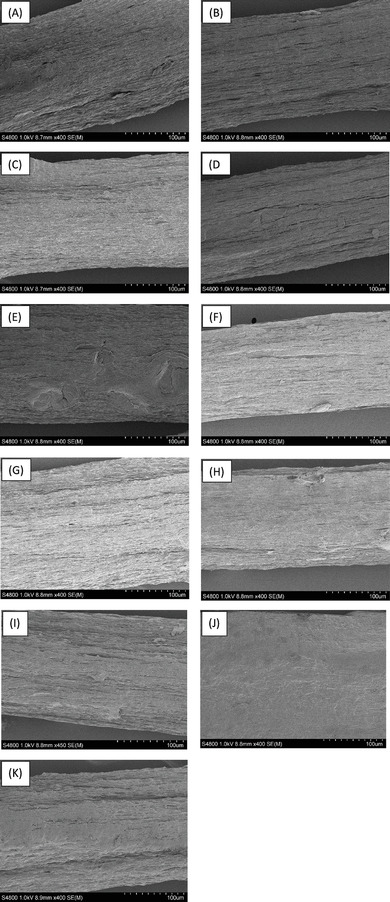
The SEM images of A) 1neg E, B) 1neg E W, C) 3neg E, D) 3neg E W, E) 5neg E, F) 5neg E W, G) 7neg E, H) 7 neg E W, I) 7neg E 1% G, J) 7neg E 2% G, K) 7neg E 3% G.

The scanning electron microscopy (SEM) images exhibit differences in the fiber morphologies after each post‐treatment. By comparing the SEM images of the monofilaments from the ethanol coagulation bath with the same monofilaments subjected to the water post treatment, the increase of orientation in the fungal microfibers as a result of the post‐treatment (**Figure** **4**A *vs*. B, C *vs*. D, E *vs*. F and G *vs*. H) can be clearly observed. Furthermore, due to drawing, the monofilaments have become thinner (Figure 4B,D,F,H). Thus, it can be argued that the orientation of the polymeric chains inside the fungal monofilaments is increased due to the water post treatment and drawing. This contention is further strengthened by the tensile test results with more than two‐fold increment of the tensile strength of water post‐treated monofilaments (Table 2). On the other hand, the monofilaments prepared with the gel that passed 7 cycles through the ultrafine grinder and were subjected to glycerol post treatments exhibited a swollen and disturbed microfiber arrangement (Figure 4I,J,K). The penetration of glycerol into the polysaccharide chains and disruption of the inter and intra molecular hydrogen bonding network could explain this disturbed morphology.^[^ ^37^ ^]^

### 2.5 Fungal Monofilaments with Tunable Properties

The tensile strength and elongation % at break data (Table 2) validate the tunability of the mechanical properties of the produced fungal monofilaments after being subjected to different post treatments.

All the monofilaments produced using the hydrogels from different grinding cycles exhibit more than a two‐fold increment in tensile strength after post treatment with water. However, the same treatment considerably reduced the elongation % at break making all the monofilaments brittle. Moreover, the increment of tensile strength after each grinding cycle indicates that the mechanical homogenization was successful without cutting the microfibers. When the 7neg monofilaments were treated with 1% glycerol, the tensile strength increased by 33% from 65 to 87 MPa. In addition to that the elongation % at break also increased by 80% from 4.0 to 7.2%. Higher glycerol concentrations resulted in formation of ductile monofilaments. With 2% and 3% glycerol treatments the tensile strength dropped to 47.6 and 30.8 MPa, respectively, and the elongation % at break increased to 11.6% and 13.8%, respectively. The drying resulted in stronger and stiffer monofilaments, which is likely explained by formation of hydrogen bonds. Recently, hydrogen bonds induced with moisture addition and compression were reported to result in increments of tensile strengths of nanocellulose films to 149.21 from 23.33 MPa^[^ ^26^ ^]^ and to 352 from 56 MPa.^[^ ^38^ ^]^ In both cases purified nanocellulose films were tested resulting in tough materials with the highest recorded toughness of 1.9 and 4.1 MJm^−3^, respectively. On the other hand, here the absorbed glycerol during the post‐treatment was retained inside the monofilament even after drying thus the plasticizing effect continued producing stretchable fibers after drying. Chen, Runge^[^ ^39^ ^]^ explain how glycerol increases the plasticizing effect in chitosan by forming single‐site hydrogen bonds that disrupt the strong hydrogen bond network in chitosan.

The highest tensile strength 140.5 MPa was achieved with the incorporation of water as the second bath. Moreover, all the monofilaments produced by using water as the second bath showed tensile strength values above 117 MPa. These are significantly higher values compared with previously produced fungal monofilament yarns by Svensson, Ferreira^[^ ^21^ ^]^ at 72.3 MPa and Svensson, Oliveira^[^ ^22^ ^]^ ≈30 to 45 MPa (P value < 0.05, assumption of sample size ≥ 3). These tensile strength values are even substantially higher than the tensile strength of 91 MPa of the wet spun monofilaments made from nano chitin hydrogels by Das, Heuser^[^ ^40^ ^]^ (P value < 0.05, assumption of sample size ≥ 3). Moreover, the 7neg E 1% G monofilaments, which were prepared with the hydrogel that had 7 grinder cycles and were post treated with 1% glycerol, also showed a higher tensile strength of 87 MPa than all the monofilaments without any post treatments with acceptable elongation % at break of 7%.

### 2.6 The Biocompatibility of the Fungal Monofilaments on Human Skin Cells

**Figure 5:**
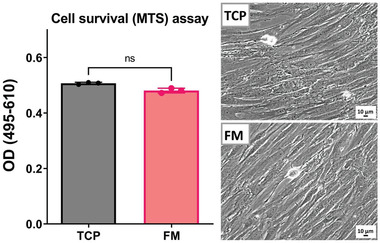
Results of MTS assay for assessment of cell mitochondrial activity. The data plotted in the graph represents the effect of fungal monofilaments on cell survival compared to control (TCP). Images show the cell density of the control sample (TCP) and the sample containing fungal monofilaments (FM).

Cytotoxicity of the fungal monofilaments on human skin cells was tested to get an overview of the biocompatibility of the monofilaments. Since the monofilaments were made using fungal biomass and when used in textile applications close contact occurs with human skin, biocompatibility is considered an important property. The results of the cell mitochondrial activity experiments done using the MTS assay show that the fungal monofilaments (FM) have no toxic effect on the proliferation and survival rate of human foreskin fibroblast (HFF) cells as there was not any significant difference between tissue culture plates (TCP) and FM groups. The phase contrast images (**Figure** **5**, TCP and FM) of the samples further confirm that the toxicity results of FM are on par with the control cell culture in TCP. According to the phase contrast images, the cells in both groups have proliferated and expanded in the same way and have become spindle‐shaped, and the order of the cells and their degree of confluence are completely the same in both groups. This indicates that the optimal growth and survival rate in the FM group is similar to what happened in the control group, so fungal monofilaments can be considered biocompatible with human skin^[^ ^23^, ^41^ ^]^

### 2.7 Material Comparison with Ashby's Plots

To obtain a clearer understanding of the monofilament mechanical properties, Ashby's material selection plots were used. With the help of these plots, clear conclusions can be obtained on which segments of conventional materials are more similar to the new material.

**Figure 6:**
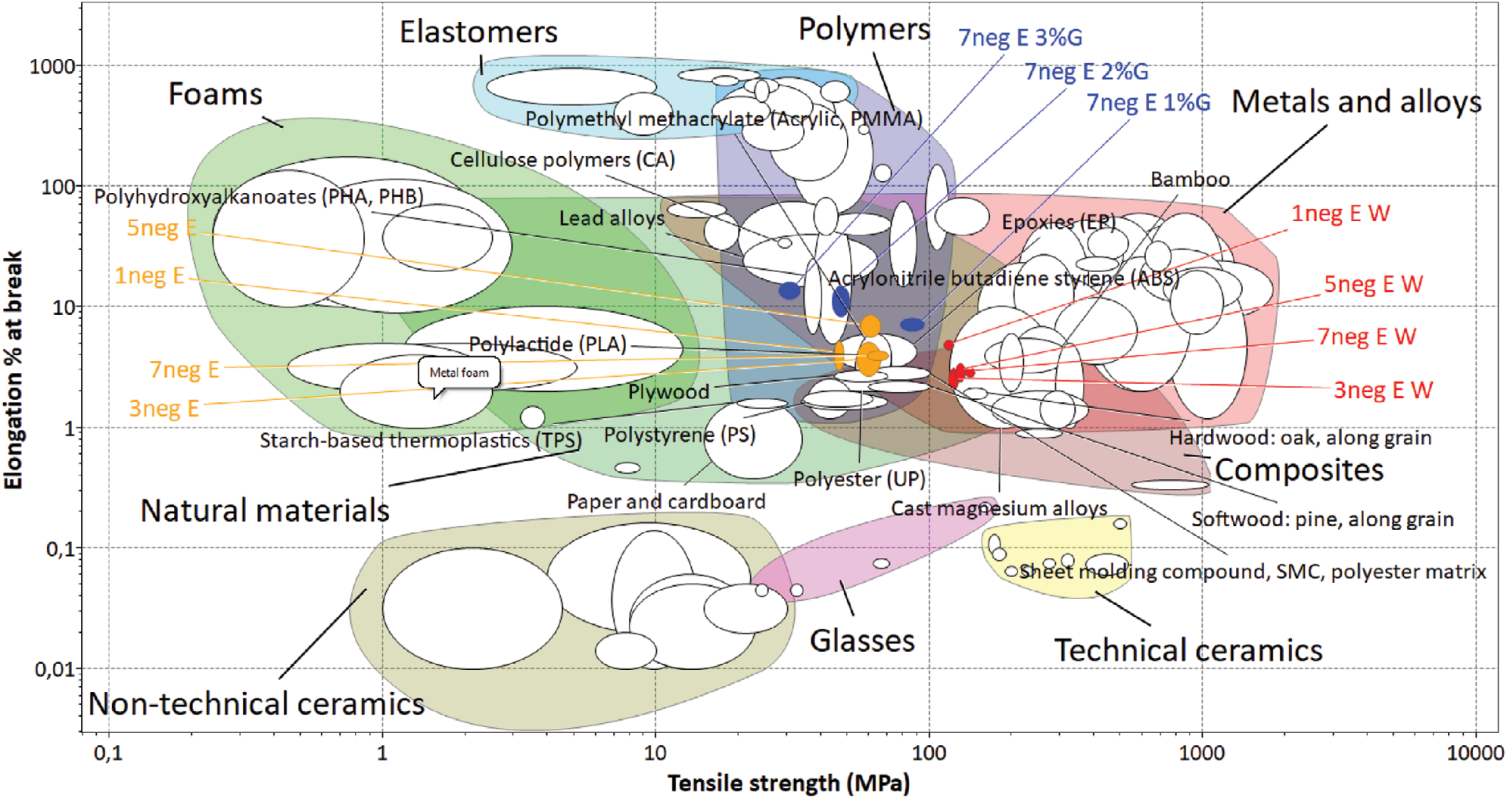
Ashby's material plot containing all general materials. (Plotted using Granta EduPack 2021 R2 Version: 21.2.0).

The Ashby plot in **Figure** **6** was plotted including the commonly known material groups using tensile strength versus elongation % at the break for the x and the y axis, respectively. All the fungal monofilaments are in the natural material bubble and the monofilaments post treated with water are more biased toward metals and alloys while untreated and glycerol post treated monofilaments are located in an area shared with the polymer bubble. When the newly derived materials are compared with the existing material it can be observed that monofilaments without any post treatments have properties close to polylactide (PLA). After post treatment with water the mechanical properties become closer to magnesium alloys and hard wood due to the increment of the tensile strength. Moreover, the properties of the monofilaments post treated with glycerol are more related to the properties of polyhydroxyalkanoates (PHA) and epoxies. The material selection graph shows how widely tunable properties were possible to obtain by using the post‐treatments of fungal monofilaments.

**Figure 7:**
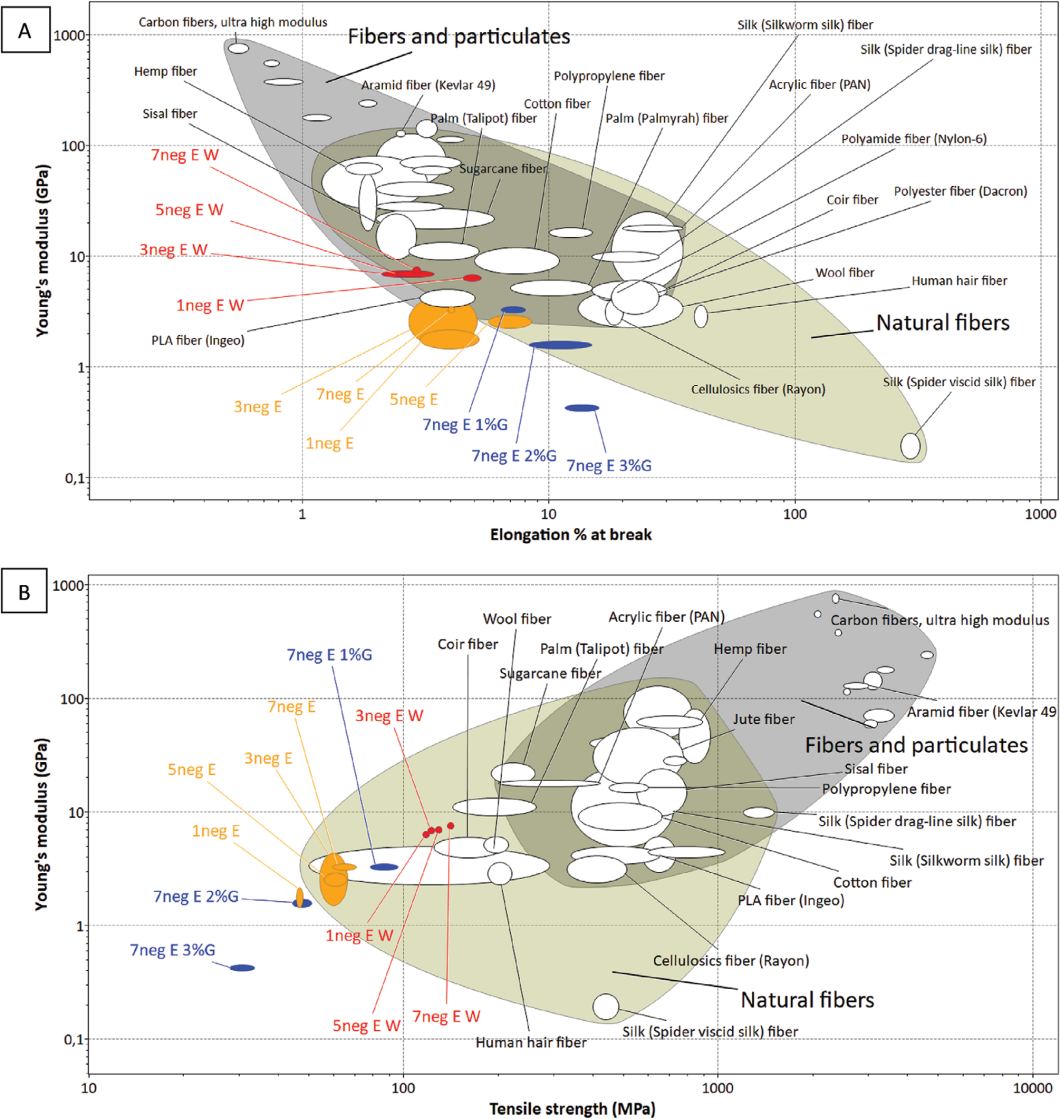
Ashby's material plot containing commercial fibers, A – Young's modulus versus elongation % at break and B – Young's modulus versus tensile strength. (Plotted using Granta EduPack 2021 R2 Version: 21.2.0).

To further relate the properties of the new fibres to already existing fibre materials two more Ashby plots were created using tensile strength, elongation % at break and Young's modulus in different combinations. In the **Figure** **7**A elongation % at break and Young's modulus has been selected as x and y axis, respectively. According to the plot, 7 monofilament types out of 11 are placed in the natural fibres bubble. Moreover, from the monofilaments which were post‐treated with water, 1neg E W shows the nearest similarity to cotton which is still the most used natural fibre in the textile industry.^[^ ^42^ ^]^ When the number of grinder cycles increased (to 3–7) the monofilaments became more similar to sisal and palm fibres (Figure 7A) which are commercially used to make rugged materials such as carpets and rattan. Even though 1neg E, 3neg E, and 7neg E monofilaments are just outside the natural fibres bubble they are almost in the same position as poly lactic acid (PLA) fibres.

Additionally, in the plot (B) of Figure 7, where tensile strength was plotted instead of elongation % at break as x axis and Young's modulus as y axis, many fungal monofilaments are shown to be closely comparable to wool fibers. After the water post treatment, the monofilaments became closer to natural plant fibers such as coir, palm, and sugarcane. However, the additional elasticity provided by the more and more increment of glycerol concentration moves the monofilaments out from the natural fibers bubble. This further confirms what was shown in the previous plot Figure 6, that higher glycerol concentration leads the monofilament toward the elastomers area. In their article Appels, van den Brandhof^[^ ^25^ ^]^ has also observed similar trend in mycelium materials with increasing glycerol concentration during the post treatments. The monofilaments after post treatment with water move toward palm and sugarcane fibers in both graphs A and B in Figure 6, thus it shows that the fungal monofilaments collected from the water second bath behaves the same way to those natural fibers regardless of tensile strength or elongation % at break plotted as the x axis.

Altogether, the produced fungal monofilaments with illustrated widely tunable properties from steel like, to polymer like, to natural fiber like showing diversified range of potential applications. The monofilaments can be used in textile applications to produce fabrics for both fashion and technical applications such as filters and membranes. The biocompatibility of the monofilaments allows usage in medical applications such as tissue engineering and wound dressings. Moreover, due to the high strength and lower flexibility in water post‐treated monofilaments, they can be used as a reinforcement layer in biobased composite material developments.

## 3 Conclusion

Monofilaments with varied mechanical properties were successfully produced from a fungus grown on food waste. The fibrous cell wall of the fungal biomass was extracted, and a hydrogel was produced. Using wet spinning method promising monofilaments were spun from the produced hydrogel. The tunability of the properties of the monofilaments by different post‐treatments was illustrated. By comparing the results with the commercially available fibers, the diversity of potential applications is also demonstrated. The fungal cultivation was done in a pilot scale and the spinning method used is already available in commercial scale for production of conventional fibers, thus the scale up of this fungal monofilament production can be easily designed in the future. Overall, the work demonstrates an alternative renewable material production using abundant waste as resource and an environment benign process with branch out promising applications.

## 4 Experimental Section

### Microorganism and Materials

The fungus *Rhizopus delemar* CBS 145940 (Centraalbureau Voor Schimmelcultures, Utrecht, The Netherlands) originally isolated from tempeh was used for valorization of bread waste. The substrate, waste bread was collected from nearby supermarkets (ICA group AB, Sweden). The bread was broken into pieces by hand and dried at room temperature for 3 to 4 days. After that, the dried bread was ground to a powder of size less than 3 mm using a rotary dry mill (M 100, Retsch Technology GmbH, Germany) and storing was done at 4 °C until use. Agar, glucose, and peptone purchased from Sigma–Aldrich were used for the preparation of agar plates. Sodium hydroxide and lactic acid obtained from Sigma–Aldrich were used for hydrogel preparation. Ethanol (absolute) acquired from VVR was used in the coagulation bath.

### Fungal Cultivation

*Rhizopus delemar* was first cultivated in agar plates containing 17.0 g L^−1^ agar, 20 g L^−1^ glucose, and 4 g L^−1^ peptone. The agar solution was prepared, and the pH was adjusted to 5.5 using 1 M NaOH and 1 M H_2_SO_4_ followed by heat sterilization using an autoclave (VX‐95, Systec, Linden, Germany) at 121 °C for 20 min. After cooling, the agar solution was poured into petri dishes. Agar plate inoculation was done using 0.1 mL of spore suspension (14 × 10^6^ spores mL^−1^; concentration was measured with a Bürker counting chamber). The incubation time of the agar plates was 3 days at 30 °C. After 3 days the agar plates were sealed with parafilm and kept at 4 °C until used.

To harvest a substantial amount of biomass, fungal cultivation was scaled up into three steps. The fungal cultivation and biomass harvesting were performed as explained in detail in our previous article,^[^ ^24^ ^]^ and briefly reported here. First, four 250 mL Erlenmeyer flasks with 100 mL of 4% bread suspension were inoculated with 2 mL of spore suspension collected from the agar plates. After 24 h, the Erlenmeyer flask cultivations were used as the pre‐culture for the 26 L bubble column bioreactor (Bioengineering, Wald, Switzerland). The reactor was steam sterilized in situ and contained 20 L of sterile 4% bread suspension. After another 24 h cultivation in the 26 L bioreactor the cultivation broth was transferred to a 1300 l airlift bioreactor (Knislinge Mekaniska Verkstad AB, Kristianstad, Sweden) which contained in situ sterilized 1000 L of 4% bread suspension. This cultivation was carried out for 48 h and the harvested biomass was washed three times by soaking in water and sieving. While washing, unconsumed substrate particles were manually removed from the biomass. Finally, the cleaned biomass was packed in plastic bags and stored at −18 °C until use. Cultivations at all three scales were performed at 35 °C.

### Alkali Treatment of Fungal Biomass

Alkali treatment on harvested fungal biomass was done using NaOH to prepare the alkali insoluble material (AIM) which mainly consists of the fungal cell wall. As the first step a suspension of biomass in water was prepared and ground once through an ultra‐fine grinder (Suzuku, Japan) by passing the solution between two MKE46 (fine silicon carbide) grinding stones for soft materials. The gap between the grinder stones and the speed were adjusted to 50 µm (open gap) and 2700 ± 50 rpm respectively. Then, sodium hydroxide and water were added to the suspension to reach a final concentration of 0.1 m NaOH and 30 g dry biomass/l. This grinding opened up the fungal hyphae and increased the NaOH penetration. Finally, the prepared biomass – NaOH suspension was heat treated in an autoclave (Systec, Germany) at 121 °C for 20 min. The AIM was separated using a 1 mm compact sieve (Russell Finex Ltd, Middlesex, UK) and washed with water until the pH became neutral.

### Preparation of Hydrogels for Wet Spinning

To prepare the hydrogel for wet spinning, first the separated AIM was diluted to 2% w/w using water and then the pH was adjusted to 4.5 using 3.5 M lactic acid. The AIM suspension was then homogenized using the same ultra‐fine grinder (Suzuku, Japan) in contact mode by grinding 1–7 cycles through −100 µm gap sizes between the MKE46 grinder discs with a rotor speed of 2700 ± 50 rpm. Samples were collected for gel preparation after 1, 3, 5, and 7 cycles. Samples labelling was done as 1neg, 3neg, 5neg, and 7neg (Table 1) respectively.

To obtain a spinning dope for wet spinning, concentration of the AIM hydrogel was required. The concentration of the gel was done using a vacuum funnel with a nylon filter membrane of 30 µm pores. Direct filtration of the AIM hydrogel collected from the grinder was not possible as the filter membrane was clogged. Dilution of the hydrogel with water was not successful as it reduced the gel homogeneity due to the pH increase. Therefore, the gel was diluted with lactic acid solution prior to filtration to maintain the homogeneity of the hydrogel with the acidic pH. For one batch, 50 g from the 2% AIM gel was mixed with 50 mL of 0.5 m lactic acid to get a homogenous diluted hydrogel that was subjected to vacuum filtration (Sterlitech, USA) to reach a gel with dry weight content of 5.5 ± 0.5%. This gel was used as the spinning dope in wet spinning process to make monofilaments.

### Hydrogel Characterization

*FluidScope scanning (oCelloScope)*: To observe the fungal microfibers and to measure the size of them after different grinding cycles a FluidScope scanning analysis was performed with the help of an oCelloScope (BioScience Solutions, Denmark). The unconcentrated AIM hydrogel samples were diluted 1:100 times using 0.5 m lactic acid to maintain the acidic pH which helps to maintain the homogeneity of the suspension. A 24 wells plate (SPL life sciences, Korea) was used with 0.5 mL of diluted suspension in each well. For each observation the illumination time was 2 ms, the imaging distance was 4.9 µm, and the number of images used was 20. To obtain an average, 50 different fungal microfiber diameter measurements were taken.

*Polarized electron microscopy (POM)*: The samples were visualized after 1, 3, 5, and 7 cycles through the ultrafine grinder, using a polarized optical microscope (POM) Nikon Eclipse LV100N POL (Japan), and the imaging software NISElements D 4.30.

### Wet spinning of Fungal Monofilaments

To form monofilaments using the concentrated hydrogel a wet spinning set up was used with ethanol as the coagulation agent according to Svensson, Ferreira.^[^ ^21^ ^]^ The wet spinning was done using a 10 mL syringe and a laboratory syringe pump (WPI, Germany). The diameter and the length of the syringe needle were 1.2 and 50 mm, respectively. The injection speed was 0.6 mL min^−1^ and the residence time of the monofilaments in the coagulation bath was 2 min. Coagulated monofilaments were fixed on a white board from the two ends using magnets for drying.

After drying, the monofilaments were soaked in water as the second bath for 30 min and again dried following the same method by fixing the two ends. Different second baths (only water and water with different concentrations of glycerol, 1%, 2%, and 3%) were tested to obtain tunable mechanical properties. After drying, the monofilaments were collected, and fiber diameter was measured using an optical microscope (Nikon. Japan) with the help of the software NIS Elements V 5.11.01 (Nikon, Japan). The details on grinding cycles, 1st and 2nd coagulation baths, and monofilament diameter correspond to the sample naming was given in Table 1.

### Characterization of the Monofilaments

*Tensile Test*: The mechanical properties of the monofilaments were measured using a tensile test equipment (H10KT, Tinius Olsen, USA) with a load cell of 100 N. The gauge length was 20 mm, the crosshead speed was 1 mm min^−1^, and the pre‐load was 0.01 N. The tensile strength and elongation % at break were obtained from the software QMat (Tinius Olsen, USA) and the Young's modulus (E) was obtained by calculating the slope of the linear part of the stress–strain curve between 0.1% to 0.5% strain. The monofilaments were pre‐conditioned overnight at 23 ± 2 °C and 50 ± 4% relative humidity (ISO 139, 2005) before the test.

*Biocompatibility test*: To check the compatibility of the produced fungal monofilaments with human skin cells, a biocompatibility test was carried out. First, human foreskin fibroblasts (HFF, Royan institute cell bank) were seeded into 96‐well culture plates (Corning, 10 452 232) at a density of 1500 cells well^−1^ with 150 µL of culture media in passage 6. Sterilized fungal monofilaments (FM) were cut into small pieces and 3 mg of them were put in cell culture inserts (96‐well plate inserts, polyester (PET) membrane 1.0 µm pore size, Corning, 10 043 832), so the final weight/volume rate was 20 mg mL^−1^. Then, loaded inserts were located into the cell‐seeded culture plates. Dulbecco's Modified Eagle's Medium (DMEM, Gibco, 11 965 092) supplemented with 10% fetal bovine serum (FBS, Gibco, 26 140 079), 2 mm L‐glutamine (Glutamax, Gibco, 35 050 061), and 100 U mL^−1^ penicillin, and 100 µg mL^−1^ streptomycin (Gibco, 15 140 122) were used for cultivating the cells at 37 °C in a humidified atmosphere of 95% air and 5% CO_2_. Cells cultured without any intervention in tissue culture plates (TCP) were assumed to be the negative control group.

The cell cultivation was carried out continuously for 7 days. On the 8th day, the culture inserts were removed, and the medium level was changed to 80 µL in each well with fresh medium. The mitochondrial activity of cells was measured using MTS/PMS reagent (CellTiter 96® AQueous One Solution Cell Proliferation Assay, Promega Corporation, G5430), which was based on the reduction of the MTS tetrazolium compound by viable mammalian cells. After adding 80 µL of fresh medium on day 8, 20 µL of MTS/PMS (3‐(4,5‐dimethylthiazol‐2‐yl)−5‐(3‐carboxymethoxyphenyl)−2‐(4‐sulfophenyl)−2H‐tetrazolium/phenazine methosulfate) reagent was added to each well. The plate was then incubated for 3 h at 37 °C in a CO_2_ incubator. The absorbance measurement was done using an ELISA microplate reader at 495 nm reference wavelength and subtracted from the absorbance at background wavelength at 610 nm (OD 495–610). All experiments were performed in triplicates.

### Scanning Electron Microscopy

The morphology of the monofilaments was observed and analyzed using an ultra‐high resolution field emission scanning electron microscope (S‐4800, Hitachi, Tokyo, Japan). A 3 kV acceleration voltage was used to obtain the images of 2 nm Pd/Pt coated fiber samples.

### Material Selection Plots

Once the mechanical properties were measured, the produced novel fibers were placed in a material selection plot to understand where they stand among the commercially used fibers. The most widespread used technique to compare materials is Ashby's bubble chart method since the graphical presentation gives an easy understanding to the properties of different materials and thus the comparison of one material to another can be effectively done.^[^ ^27^, ^43^ ^]^ To plot the graphs Granta Edupack 2021 R2 version: 21.2.0 (Ansys Inc. USA) with level 3 advanced material database was used. Different mechanical properties were used as x‐and y‐axis. The comparison of novel fibers was done with the help of the graphical data.

### Statistical Analysis of Data

The number of data replicates were either equal or more than 3 in all instances. The data was analyzed using Minitab 21 (Minitab 21.1.1) software and the significance between two mean values was tested using 2 sample *t* test with 95% probability.

## Conflict of Interest

The authors declare no conflict of interest.

## Supporting information

Supporting Information

## Data Availability

The data that support the findings of this study are available from the corresponding author upon reasonable request.

## References

[1] Unece , UN Alliance aims to put fashion on path to sustainability, in: Europe UNECf (ed.) 2018, https://unece.org/forestry/press/un-alliance-aims-put-fashion-path-sustainability (accessed: March 2023).

[2] Unfcc , UN Helps Fashion Industry Shift to Low Carbon, in: change Unc (ed.) 2018, https://unfccc.int/news/un-helps-fashion-industry-shift-to-low-carbon (accessed: April 2023).

[3] K. Niinimäki , G. Peters , H. Dahlbo , P. Perry , T. Rissanen , A. Gwilt , Nat. Rev. Earth & Environ. 2020, 1, 189.

[4] T. Karthik , R. Rathinamoorthy , Handbook of Ecomaterials, Springer, Basel 2018, pp. 1–27.

[5] C. P. Jiménez‐Gómez , J. A Cecilia , Molecules 2020, 25, 3981. 32882899 10.3390/molecules25173981 PMC7504732

[6] W. M. F. B. W. Nawawi , M. Jones , R. J. Murphy , K.‐Y. Lee , E. Kontturi , A. Bismarck , Biomacromolecules 2020, 21, 30. 31592650 10.1021/acs.biomac.9b01141 PMC7076696

[7] J. Sebastian , T. Rouissi , S. K Brar , in Handbook of Chitin and Chitosan (Eds: S. Gopi , S. Thomas , A. Pius ), Elsevier, Amsterdam 2020, pp. 419–452.

[8] A. Zamani , M. J Taherzadeh , Iranian Poly. J. 2012, 21, 845.

[9] S. Kaur , G. S. Dhillon , Crit. Rev. Microbiol. 2014, 40, 155. 23488873 10.3109/1040841X.2013.770385

[10] S. C. Tan , T. K. Tan , S. M. Wong , E. Khor , Carbohydr. Polym. 1996, 30, 239.

[11] R. Gmoser , J. A. Ferreira , P. R. Lennartsson , M. J Taherzadeh , Fungal Bio. Biotechnol. 2017, 4, 4. 28955473 10.1186/s40694-017-0033-2 PMC5611665

[12] R. Gmoser , R. Fristedt , K. Larsson , I. Undeland , M. J. Taherzadeh , P. R Lennartsson , Bioengineered 2020, 11, 582. 32449450 10.1080/21655979.2020.1768694 PMC8291841

[13] A. Sadaf , S. Kumar , L. Nain , S. K. Khare , Biocat. Agricul. Biotechnol. 2021, 32, 01934.

[14] P. F. Souza Filho , R. B. Nair , D. Andersson , P. R. Lennartsson , M. J Taherzadeh , Fungal Bio. Biotechnol. 2018, 5, 5. 29619233 10.1186/s40694-018-0050-9 PMC5880086

[15] D. Troiano , V. Orsat , M.‐J Dumont , Appl. Microbiol. Biotechnol. 2022, 106, 4029. 35608668 10.1007/s00253-022-11984-1

[16] Unep , Food Waste Index Report 2021, 100.

[17] A. S. Demirci , I. Palabiyik , T. Gumus , J. Biotechnol. 2016, 231, S13.

[18] N. Struyf , E. Maelen , S. Hemdane , J. Verspreet , K. Verstrepen , C. Courtin , Comprehen. Rev. Food Sci. Food Safety 2017, 16, 850. 10.1111/1541-4337.12282 33371607

[19] M. A. Naranjo‐Ortiz , T. Gabaldón , Biological Rev. 2019, 94, 2101. 10.1111/brv.12550 PMC6899921 31659870

[20] Y. Tominaga , Y. Tsujisaka , Agric. Biol. Chem. 1981, 45, 1569.

[21] S. E. Svensson , J. A. Ferreira , M. Hakkarainen , K. H. Adolfsson , A. Zamani , Sustain. Mater. Technol. 2021, 28, e00256.

[22] S. E. Svensson , A. O. Oliveira , K. H. Adolfsson , I. Heinmaa , A. Root , N. Kondori , J. A. Ferreira , M. Hakkarainen , A. Zamani , Int. J. Biol. Macromol. 2022, 209, 618. 35427640 10.1016/j.ijbiomac.2022.04.031

[23] M. Benedikt Maria Köhnlein , T. Abitbol , A. Osório Oliveira , Mater. Des. 2022, 216, 110534.

[24] K. Wijayarathna , G. Mohammadkhani , A. M. Soufiani , K. H. Adolfsson , J. A. Ferreira , M. Hakkarainen , L. Berglund , I. Heinmaa , A. Root , A. Zamani , Resour., Conserv. Recycl. 2022, 179, 106041.

[25] F. V. W. Appels , J. G. van den Brandhof , J. Dijksterhuis , G. W. de Kort , H. A. B. Wösten , Commun. Bio. 2020, 3, 334. 32591629 10.1038/s42003-020-1064-4 PMC7320155

[26] X. Han , Z. Wang , L. Ding , L. Chen , F. Wang , J. Pu , S. Jiang , Chin. Chem. Lett. 2021, 32, 3105.

[27] M. F. Ashby , D. Cebon , Le J. de Physique IV 1993, 3, C7.

[28] N. M. Stark , L. M Matuana , Mater. Today Sustain. 2021, 15, 100084.

[29] M. D. Marhendraswari , K. Mondylaksita , R. Millati , Mater. Sci. Engineer. 2020, 991, 12041.

[30] X. Zhou , J. Sethi , S. Geng , L. Berglund , N. Frisk , Y. Aitomäki , M. M. Sain , K. Oksman , Mater. Des. 2016, 110, 526.

[31] Z. Yaneva , D. Ivanova , N. Nikolova , M. Tzanova , Biotechnol. Biotechnol. Equip. 2020, 34, 221.

[32] S. Botelho da Silva , M. Krolicka , L. A. M. van den Broek , A. E. Frissen , C. G. Boeriu , Carbohydr. Polym. 2018, 186, 299. 29455991 10.1016/j.carbpol.2018.01.050

[33] T. Zhong , M. P. Wolcott , H. Liu , J. Wang , J. Cleaner Prod. 2020, 250, 119458.

[34] J. Huaytragul , J. Chalitangkoon , P. Monvisade , N. Chotsaeng , J. Taiwan Inst. Chem. Eng. 2021, 123, 293.

[35] I. A. Finneran , P. B. Carroll , M. A. Allodi , G. A Blake , Phys. Chem. Chem. Phys. 2015, 17, 24210. 26325657 10.1039/c5cp03589a

[36] J. Tarique , S. M. Sapuan , A. Khalina , Sci. Rep. 2021, 11, 13900. 34230523 10.1038/s41598-021-93094-y PMC8260728

[37] J. Rodríguez , T. J. Madera‐Santana , D. Sánchez‐Machado , J. Lopez‐Cervantes , H. Valdez , J. Polym. Environ. 2014, 22, 41.

[38] X. Han , Y. Ye , F. Lam , J. Pu , F. Jiang , J. Mater. Chem. A 2019, 7, 27023.

[39] M. Chen , T. Runge , L. Wang , R. Li , J. Feng , X.‐L. Shu , Q.‐S. Shi , Carbohydr. Polym. 2018, 200, 115. 30177147 10.1016/j.carbpol.2018.07.062

[40] P. Das , T. Heuser , A. Wolf , B. Zhu , D. E. Demco , S. Ifuku , A. Walther , Biomacromolecules 2012, 13, 4205. 23102411 10.1021/bm3014796

[41] K. B. Narayanan , S. M. Zo , S. S Han , Int. J. Biol. Macromol. 2020, 149, 724. 32004611 10.1016/j.ijbiomac.2020.01.276

[42] M. Tausif , A. Jabbar , M. S. Naeem , A. Basit , F. Ahmad , T. Cassidy , Textile Progress 2018, 50, 1.

[43] D. U. Shah , Mater. Des. 2014, 62, 21.

