# Supplementary material for: Tunable Fungal Monofilaments from Food Waste for Textile Applications
Source: Glob Chall. 2023 Sep 17;8(3):2300098. doi: 10.1002/gch2.202300098 (PMC10935878; doi:10.1002/gch2.202300098)
Supplement: Supplementary file 1 — Supporting Information [file GCH2-8-2300098-s001.pdf]

Global
Challenges
Open Access

Supporting Information

for *Global Challenges.*, DOI 10.1002/gch2.202300098

Tunable Fungal Monofilaments from Food Waste for Textile Applications

E. R. Kanishka B Wijayarathna, Ghasem Mohammadkhani, Farshad Homayouni Moghadam, Linn Berglund, Jorge A. Ferreira, Karin H. Adolfsson, Minna Hakkarainen and Akram Zamani**

Supporting information file - Tunable fungal monofilaments from food waste for textile applications

E R Kanishka B Wijayarathna[1], Ghasem Mohammadkhani[1], Farshad Homayouni Moghadam[2], Linn Berglund[3], Jorge A. Ferreira[1], Karin H. Adolfsson[4], Minna Hakkarainen[4], Akram Zamani[1]

[1] Swedish Centre for Resource Recovery, University of Borås, Borås SE-501 90, Sweden

[2] Department of Animal Biotechnology, Cell Science Research Center, Royan Institute for Biotechnology, ACECR, Isfahan, Iran

[3] Department of Engineering Sciences and Mathematics, Luleå University of Technology, Luleå SE-971 87, Sweden

[4] Department of Fiber and Polymer Technology, KTH Royal Institute of Technology, Stockholm SE-100 44, Sweden

1. A pair of 20 cm monofilament samples underwent the post treatments and ca 1 cm of drawing was added when the monofilaments were fixed to drying. Depending on the observation the amount of drawing was finalized as 5%.

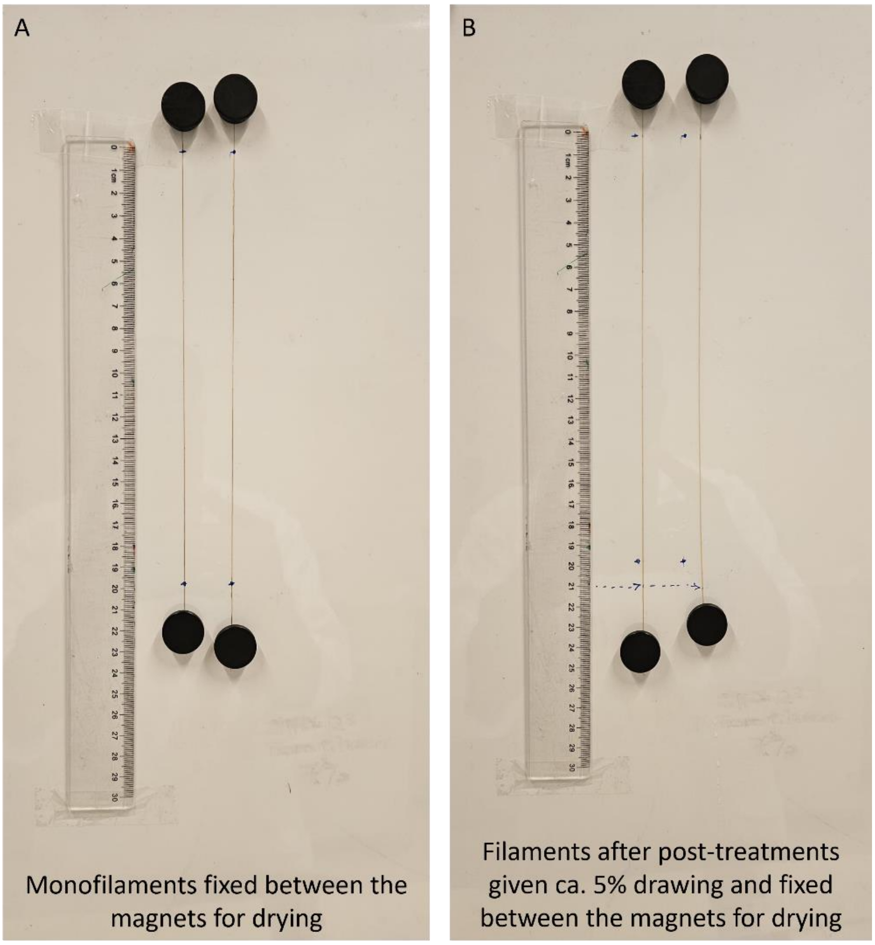

Figure S1: How drawing was added to the fungal monofilaments when drying after the post treatments
